# Deletion of chr7p22 and chr15q11: Two Familial Cases of Immune Deficiency: Extending the Phenotype Toward Dysimmunity

**DOI:** 10.3389/fimmu.2019.01871

**Published:** 2019-08-16

**Authors:** Natacha Sloboda, Arthur Sorlin, Mylène Valduga, Mylène Beri-Dexheimer, Claire Bilbault, Fanny Fouyssac, Aurélie Becker, Laëtitia Lambert, Céline Bonnet, Bruno Leheup

**Affiliations:** ^1^Clinic Genetics Department, Children Hospital, CHRU Nancy, Nancy, France; ^2^Genetics Laboratory, CHRU Nancy, Nancy, France; ^3^Infantile Medicine Department (Neuropediatrics), Children Hospital, CHRU Nancy, Nancy, France; ^4^Infantile Medicine Department (Hematopediatrics), Children Hospital, CHRU Nancy, Nancy, France

**Keywords:** 7p22, vasculitis, hypogammaglobulinemia, CGH array, dysimmunity

## Abstract

**Background:** We report here two new familial cases of associated del15q11 and del7p22, with the latter underlining the clinical variability of this deletion. Two siblings patients presented a similar familial imbalanced translocation, originating from a balanced maternal translocation, with deletions of 7p22 and of 15q11 [arr[GRCh37] 7p22.3-p22.2(42976-3736851)x1, 15q11.1-q11.2(20172544-24979427)x1].

**Methods:** We used aCGH array, FISH, and karyotype for studying the phenotype of the two patients.

**Results:** The 7p22 deletion (3.5 Mb) contained 58 genes, including several OMIM genes. Patients 1 and 2 exhibited acquisition delays, morphological particularities, and hypogammaglobulinemia, which was more severe in patient 1. Patient 1 presented also with cerebral vasculitis.

**Conclusion:** We discuss here how the PDGFa, CARD11, LFNG, GPER1, and MAFK genes, included in the deletion 7p22, could be involved in the clinical and biological features of the two patients.

## Case Presentation

We report here two new familial cases of del7p22 which underline the clinical variability of this deletion. The patient's legal representatives accepted the use of medical data for research purposes. They signed consent forms from the Clinical Genetics Department of the Nancy University Hospital in accordance with French regulations. The patient's legal representatives signed also consent forms for the publication of this case report and any potentially identifying images and information.

Chromosome analyses were performed by means of GTG banding (G-banding using trypsin and Giemsa stain). Chromosome abnormalities were described according to the International System for Human Cytogenetic Nomenclature (ISCN). FISH was performed on metaphase chromosomes and interphase nuclei under standard conditions by using specific DNA probes for chromosome 7 telomeres, namely 7p 7q Vysis telomeric probes, and for the Prader–Willi–Angelman Critical Region (PWACR). Analysis of der15 was performed using Kreatech probe SE15D15Z4. For metaphase chromosomes, the locus was considered to be duplicated when a consistently brighter signal in one chromosome 15 was seen compared to the one of the homolog chromosome. Examination of interphase nuclei completed the observation and a positive result was established when three fluorescent spots were observed.

Cytogenetic comparative genomic hybridization array (aCGH) analyses were performed from leukocytes of heparin-treated peripheral blood. Genomic DNA was extracted from leukocytes of EDTA-treated peripheral blood using a manual procedure (Nucleon BACC3, Amersham Biosciences, GE Healthcare Europe, Orsay, France). DNA concentration and purity were determined by measuring UV absorbance at 260 and at 280 nm. A aCGH was performed with the Agilent kit 244 A (Agilent Technologies, Santa Clara, CA) according to manufacturer's instructions. Breakpoint positions were reported according to build 37, Hg 19.

Patient 1 is a Caucasian male who was diagnosed with hypogammaglobulinemia M. Relative leucopenia (4,058 cells/μL leucocyte, normal range 4,000–10,000) and iron deficiency anemia (Hb 7.7 g/dL, Fe 7 mmol/L) were reported at 1 month of age. He had a history of poor food intake since 3 months of age, with associated food difficulties, resulting in transient weight loss (4,860 g (−3 SD) at 4 months, 5,400 g (−3 SD) at 5 months, 7,020 g (−2 SD) at 8 months, and back to the average at 14 months at 1,1260 g). Stature growth remains between −1.5 SD of the mean at the same time.

Regarding development, he had delays in speech and in language, but not in gross and fine motor skills: sitting was acquired at 12 months, walking at 14 months. Motor development was within the normal range at 3 years. The speech delay (3 words at 20 months) was partially attributed to oral-facial difficulties (intermittent drooling, sucking difficulties from birth, refusal of feeding in pieces). An adapted treatment by a speech therapist allowed progress over a few years, however, at 8 years old, he was unable to read and calculate.

**Figure 1:**
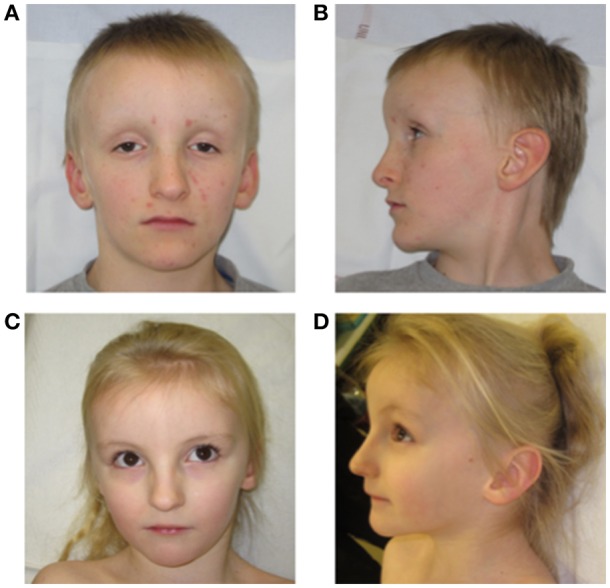
Face **(A)** and side **(B)** of the patient 1 at 8 years old; Face **(C)** and side **(D)** of the patient 2 at 5 years old.

On morphological examination, there was only a triangular face in the neonatal period. At 8 years old, distinctive features were noted: bilateral deep-set eyes, high nasal origin and thin nasal alae, prominent forehead with bossing, large ears, a triangular face, short philtrum, thin upper lip, and a discrete inverted lower lip. He is a very blond child with pale complexion and thin skin (Figures 1A,B).

The patient presented a significant number of infectious episodes during his first years of life, including two hospitalizations during the first year of life for hyperthermia from probable viral diseases, and at 1 year old a hospitalization for a bilateral otitis (pneumococcus with intermediate sensitivity). Numerous acute otitis media infections in childhood caused the installation of trans-tympanic drains at 3 years old; there were also many asthmatic bronchitis attacks in winter, without established treatment. At 8 years old, he developed a varicella. At 8 years and 4 months, he was seen at the emergency department due to fainting, with recurrent left facial paralysis, motor deficiency in the left upper limb and ataxia, which spontaneously resolved. The MRI examination revealed ischemia in the lenticular and striated nuclei associated with bilateral sub cortico-frontal hypersignals, in favor of a cerebral vasculitis, with repeated transient ischemic attacks.

Investigation of this vasculitis revealed an immune deficiency (summarized in Table 1), with a neutropenia at 1,120 cell/μL (reference range 1,500–8,000 cell/μL), an hypogammaglobulinemia G (6.72 g/L, reference range 8.29–14.19 g/L), A (0.38 g/L, reference range 0.71–1.91 g/L), and M (<0.22 g/L, reference range 0.46–1.12 g/L) and a decrease in T (681 cell/μL, reference range 700–4,200 cell/μL), and NK lymphocytes (79 cell/μL, reference range 90–900 cell/μL). Several post-vaccinia antibodies were weakly positive [IgG anti-tetanic 0.09 UI/ml (reference range > 0.1); IgG anti-diphteric 0.85 UI/ml, reference range for complete protection >1.000]. The lymphocyte B subpopulations showed a deficiency in B memory cells (CD19+ CD27+, 6%, (18.6–46.7%) and in switched B cells (CD19+ CD27+ IgD- 0.3%, (10.9–30.4%). An extended septic workup was performed. This excluded parvovirus B19, hepatitis A, hepatitis B, hepatitis C, cytomegalovirus, C. pneumonia, Herpes Simplex virus type 1, 2, and 6 infections. Noteworthy, we found a past infection for varicella (ELISA IgG Index 2,000 U/mL and ELISA IgM index 0.038 U/mL). Analysis of the cerebrospinal fluid was performed during the vasculitis episode. The results were normal for chemistry (lactates, chlorides, proteins, carbohydrate); Germ culture was negative. PCRs for HSV, EBV, and CMV were negative. Anti-borrelia IgM and IgG were also negative. Vasculitis may be secondary to VZV infection, as postvaricella angiopathy is the most common infectious/inflammatory cause of vascular stroke in children (2), and corresponds to a vascular stroke occurring within 12 months of VZV infection (3). Even though PCR for VZV was negative, these elements remain compatible with the clinical situation of sibling 1. Indeed, when VZV PCR is negative, the presence of VZV antibodies in the CSF can be an argument to prove VZV infection (4). Unfortunately, the detection of these antibodies was not performed during the vasculitis episode.

**Table 1:** Biological parameters.

	**Patient 1**							**Patient 2**			
**Cell count**	**Normal range**	**8 years 4 months**	**9 years 5 months**	**10 years 5 months**	**11 years 5 months**	**12 years 5 months**	**13 years 5 months**	**3 years 8 months**	**4 years 8 months**	**5 years 8 months**	**6 years 8 months**
Lymphocytes (cells/μL)	1,500–6,500	1,030	1,170	1,350	1,680	1,550	1,410	2,190 *(3,000–9,500)*	1,540 *(2,000–8,000)*	2,830 *(2,000–8,000)*	2,900 *(1,500–7,000)*
PNN (cells/μL)	1,500–8,000	↓**1,120**			2,580	2,890	1,760	4,300	3,210	9,770	4,190
NK (CD3-/CD16_56+) (cells/μL)	90–900	↓**79**		143					165	803	628
LT (CD3+) (cells/μL)	700–4,200	↓**681**		930					1,506	1,453	1,643
LB (CD19+)	200–1,600	207							372	563	480
LB CD19+ CD27+ (memory) %		↓**6** *(18.6–46.7*)		↓**5** *(18.6–46.7*)					12 *(7.8–37.1*)		8 *(18.6–46.7*)
LB CD19+ CD27+ (memory) (cells/μL)		↓**39** *(60–230*)		↓**9** *(60–230*)					45 *(50–390*)		27 *(60–230*)
LB CD19+ CD27- IgD+ (naives) %		↓**22.6** *(47.3–77*)		90.5 *(47.3–77*)							86 *(47.3–77*)
LB CD19+ CD27- IgD+ (naives) (cells/μL)		581 *(130–460*)		158 *(130–460*)							313 *(130–460*)
LB CD19+ CD27+ IgD+ (marginal) %		↓**0.30** *(5.2–20.4*)		↓**3.50** *(5.2–20.4*)					6.5 *(2.7–19.8*)		↓**3** *(5.2–20.4*)
LB CD19+ CD27+ IgD+ (marginal) (cells/μL)		↓**8** *(20–100*)		↓**6** *(20–100*)					24 *(20–180*)		↓**11** *(20–100*)
LB CD19+ CD27+ IgD- (switched) %		↓**0.30** *(10.9–30.4*)		↓**2.3** *(10.9–30.4*)					7.2 *(4.7–21.2*)		6 *(10.9–30.4*)
LB CD19+ CD27+ IgD- (switched) (cells/μL)		↓**8** *(40–140*)		↓**4** *(40–140*)					27 *(20–220*)		22 *(40–140*)
Ig G (g/L)		↓**6.72** *(8.29–14.19*)	↓**7.15** *(8.29–14.19*)	↓**7.83** *(8.29–14.19*)	↓**8.16** *(6.90–14.00*)	7.23 *(6.90–14.00*)	↓**6.8** *(6.90–14.00*)	↓**6.11** *(7.01–11.57*)	↓**6.34** *(6.67–11.79*)	8.91 *(6.67–11.79*)	7.23 *(6.90–14.00*)
Ig A (g/L)		↓**0.38** *(0.71–1.91*)	↓**0.51** *(0.71–1.91*)	↓**0,38** *(0.71–1.91*)	↓**0.46** *(0.70–4.10*)	↓**0.36** *(0.70–4.10*)	↓**0.31** *(0.70–4.10*)	↓**0.34** *(0.66–1.20*)	↓**0.44** *(0.79–1.69*)	↓0.62 *(0.79–1.69)*	↓**0.36** *(0.70–4.10*)
Ig M (g/L)		↓** <0.22** *(0.46–1.12*)	↓** <0.22** *(0.46–1.12*)	↓** <0.22** *(0.46–1.12*)	↓** <0.22** *(0.40–2.40*)	↓** <0.22** *(0.40–2.40*)	↓** <0.22** *(0.40–2.40*)	↓**0.23** *(0.38–0.74*)	↓**0.33** *(0.40–0.9*)	↓0.23 *(0.40–0.9*)	↓** <0.22** *(0.40–2.40*)
**Serology**											
IgG Measles (Elisa index)		500,000 (+)							7,000 (+)		
IgG Measles (UA/mL)	>16.5 UA/mL						26.5				
IgG Mumps (Elisa index)		<230,000 (–)							<230 (–)		
IgG Oreillons (UA/mL)	>16 UA/mL						6				
IgG Rubella (UI/mL)	>15 UI/mL	5					1.6		51		
IgG anti-tetanus (UI/mL)	cf. Legend	0.09	0.22	0.08		0.5	0.18		0.27	0.26	0.5
IgG anti-diphtheria (UI/mL)	cf. Legend	0.85							0.45	0.26	
IgG anti-hemophilic (μg/mL)	>1 μg/mL	1.19 μg/mL			>9	>9					
IgG anti pneumococcus (μg/mL)	cf. Legend	32.1			22.5	28					

*Normal ranges for Lymphocytes, PNN, NK, LT, LB, and serology are indicated in the left most column of the table. Normal ranges variating with age are indicated in (italics between brackets), below the measured values. Values outside the accepted physiological range are reported in bold, with an arrow indicating when values are too low. Lymphocytes, PNN, NK, LT, LB, IgG, IgA and IgM, and serology range values are the ones routinely used in Nancy University Hospital. B lymphocyte subpopulations range values were found in the work of Piatosa et al. (1). Anti-tetanus IgG (UI/mL): <0.01 UI/mL: No efficient protection. Complete vaccination recommended; 0.010–0.09 UI/mL: Weak antibody titer, no efficient protection Booster recommended; 0.10–0.49 UI/mL: Efficient protection, but booster advisable; 0.5–0.99 UI/mL: Efficient protection. Booster after 2 years. Anti-diphteria IgG: <0.01 UI/mL: No efficient protection. Complete vaccination recommended; 0.010–0.09 UI/mL: Weak antibody titer, no efficient protection Booster recommended; 0.10–1.0 UI/mL: Efficient long term protection. Booster after 5 years. Anti-pneumococcus IgG: No threshold. Observed mean of the normal patient around 50 μg/mL*.

The rest of the balance, including Holter ECG, cardiac, and supra-aortic trunk ultrasound recordings were without abnormalities.

Treatment was initiated with Enoxaparin, which was replaced by Acetylsalicylic acid at an anti-aggregating dose (100 mg/day) with the normal cardiac workup. Corticosteroid treatment was initiated secondarily, at the dose of 1.2 mg/kg/day for a period of 3 months, to be reduced according to the MRI data and the opinion of the neuro-pediatrician. The treatment was stopped 2 years later. No immunological supplementation treatment has been instituted yet. Regular monitoring (once a year in the absence of atypical infection) is applied, including assays of vaccine serology (vaccine booster if necessary) and immunoglobulin assays. There has been no other episode of vasculitis or other serious disorders since then.

Patient 2, sister of patient 1, was born in 2011. She carries the same chromosomal imbalance, diagnosed during pregnancy, and confirmed by a caryotype and a FISH during the post-natal period. She is a Caucasian female and like her brother, she had delays in speech and in language, but not in gross and fine motor skills, and she began to walk at 16 months old. She has also a history of poor food intake during her firsts months of life, without severe failure to thrive [4.8 kg (−3 SD) and 76 cm (−1.5 SD) at 22 months; 12 kg (−1 SD) and 97 cm (+1.3 SD) at 3 years].

On physical examination, she exhibits very similar features compared with her brother (shape of the face, forehead, nose appearance; Figures 1C,D).

In this familial context, she was monitored earlier, due to the vasculitis episode that occurred in her brother. Her evolution is more favorable. Parents did not report atypical infections. She had some asthmatic bronchitis and several episodes of nasopharyngitis per year, without fever or complications. Her immunity evaluation showed a moderate decrease in immunoglobulins G, A and M, with respective values of 6.11 g/L (reference range 6.67–11.79 g/L), 0.34 g/L (reference range 0.66–1.20 g/L), and 0.23 g/L (reference range 0.38–0.74 g/L). This hypogammaglobulinemia G and M was similar to her brother's; she presented also with a negative post-vaccine serology (anti-tetanic and anti-diphteric), and a normal distribution of subpopulations of B lymphocytes. The post-vaccine serology was monitored once a year, and an appropriate vaccine booster was performed when the vaccine protection was too weak.

The absence of atypical or severe infections in patient 2 is an argument for keeping to simple monitoring.

**Figure 2:**
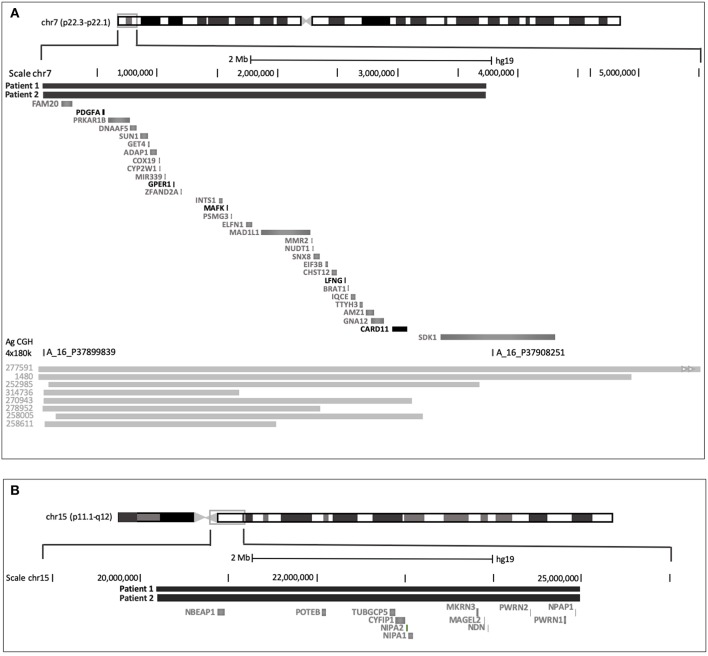
**(A)** Deleted region of the chromosome 7 in patients 1 and 2. The deletion (thick black line) in patient 1 overlaps by 85% the deletion described in the decipher patient n°1,480 (several decipher patients with high overlap in gray line). The Agilent aCGH probes are shown. SDK1 is truncated by the deletion (chromosomic break point), and all OMIM genes are lost in the deletion. The gene of interest are in black. **(B)** Deleted region of the chromosome 15 in patient 1 and 2. Different OMIM and RefSeq Genes are listed. The deletion (thick black line) does not affect the PW and AS sites.

Patients 1 and 2 were described with clinical immune deficiency, intellectual disability, or multiple congenital anomalies referred for aCGH. Analyses of Patient 1 revealed two deletions, formula arr[GRCh37] 7p22.3-p22.2(42976-3736851)x1, 15q11.1-q11.2 (20172544-24979427)x1: a 3.69 Mb deletion at 7p22 and a 4.8 Mb deletion at 15q11. These copy number variants (CNVs) result from an imbalance of a maternal translocation involving chromosome 7 and 15 with loss of the derivative of chromosome 15 (Figure 2). Thus, the chromosomic formula for patient 1 is 46, XY, t (5, 6) (p22; q12) del der15.

In patient 2, aCGH analysis (confirmed by FISH) shows the formula arr[GRCh37]7p22.3p22.2(65558_3736851)x1,15q11.2 (22765628_24979427)x1.

The formulas differ slightly as the terminal deletion of chromosome 7 varies from 1 probe at the telomeric end (probably due to the variability of the technique and the coverage of the end of the chromosome), but the interstitial end is strictly identical. There is a variation of several probes on chromosome 15, but this is due to a polymorphism of the control for patient 1; the disequilibrium bounds of variation are therefore probably the bounds in patient 2. Despite these variations, we could conclude that Patients 1 and 2 inherited the same genetic imbalance, with the same genes missing.

## Background

To date, DECIPHER referenced 83 patients (34 patients with loss of material) with an overlap of this 7p22 region. Clinical presentation was available for 26 patients: 13 presented with cognitive impairment or intellectual disabilities. Only one patient (DECIPHER *n*°1,480), holds an 85% overlap with our patient, exhibits features such as T lymphocytopenia, cutaneous abnormalities, cutaneous photosensitivity, microcephaly, and delayed acquisition. This patient has also a more important imbalance arising from a balanced parental rearrangement than for patient 1 (4.96 Mb, chr7:1–4, 962, 159). This deletion removes fully the Sidekick Cell Adhesion Molecule 1 gene (SDK1) while, the last half of SDK is still present in our Patients (Figure 2).

The deleted 7p22 region spans 58 genes, including some that could be linked with the clinical signs of patient 1. There is also a large sequence overlap with patients 4 and 5 of Yu and colleagues' case report (7), which is similarly dysmorphic, with no reported hematologic manifestations. In this study, the patients displayed weaknesses in language skills, as well as in motor skills. The characteristic facial features described included a broad nasal root, a prominent forehead, a prominent glabella, and arched eyebrows. Other variable features were highlighted, such as microcephaly, metopic ridging or craniosynostosis, cleft palate, cardiac defects, and mild hypotonia. Noteworthy, our patients do not have any cardiac or cranial abnormalities.

## Discussion

We have used the predictive pLI score to discuss the role of the different genes, as this score evaluates the probability for a gene to be intolerant to a loss of function (LoF) mutation (8). Briefly, the score is calculated using the following ratio: observed LoF variants/expected LoF variants in ExAC. The pLI was also described by Gambin et al. (9) as very well correlating with the haploinsufficiency prediction score set up by Huang et al. (5). As this haploinsufficiency score is a computational prediction, we preferred to use the pLI score to discuss our observations. The closest from 1 is the pLI score, the highest are the chances that a LoF mutation (and to a certain extent, haploinsufficiency), is causing the disease.

The deletion detected at 15q11.1-q11.2 with the aCGH showed that 106 genes are contained in this deletion including several OMIM genes, as well as NBEAP1, responsible for B cell leukemia in translocation events (6, 10), and NDN, belonging to the contiguous genes responsible for the Prader Willi syndrome. In the case of our patients, the loss of chromosomal material on chromosome 15 is unrelated to the Prader Willi/Angelman region (Figure 2B, the SNRPN region and UBE3A are not included in the deletion). Different databases (Decipher, ClinGen, DGV) have recorded several hundreds of patients with overlaps, including a hundred with intellectual disability, but no patients so far were described with either cellular or humoral immune deficiency. Taken together, these data suggest that the deletion 15q11.1-q11.2 is probably not involved in the phenotype of patient 1 and 2.

Immune deficiency may be related to several genes, especially Platelet-derived growth factor alpha (PDGFalpha), caspase recruitment domain 11 (CARD11), N-acetylglucosaminyltransferase lunatic fringe (LFNG), MafK, and G protein-coupled estrogen receptor 1 (GPER-1).

PDGFalpha was reported to synergize with Interferon gamma to induce cxcl10 production in macrophages (11). According to the ExAc database, it is rather intolerant to haploinsufficency (pLI = 0.86). PDGFalpha has not yet been associated with a disease.

CARD11 has been linked to severe combined immunodeficiency (pLI = 1.00). That was the case in patients with homozygous mutations, leading to a severe phenotype spanning from increased transitional B cell counts (12) to increased naive and transitional B cell counts (13).

CARD11 has also been described by Snow et al. (14) in a man and his 2 daughters, with increased transitional B cell counts, with NFKB, and T-cell anergy. This family presents a heterozygous mutation [c.735G-A transition in exon 5, resulting in a glu127-to-gly (E127G) substitution] causing a LoF of CARD11. Therefore, haploinsufficiency could lead to decreased Ig levels. Even though none of these studies included Ig level measurements, the LoF of CARD11 in heterozygous mutant mice resulted in a complete blockade of B cell activation (15). This result is in line with recent studies where heterozygous hypomorphic CARD11 mutations induce features such as hypogammablobulinemia and an increased immature B cell count (6, 10, 16).

CARD11 is also required for efficient development of NK cells and B cells in mice *in vivo* (17), as well as for B cell proliferation [through its CARD domain (15), and activation (18). Antibody response titer was also reduced in mice sera when CARD11 was mutated in its coiled-coil region, resulting in diminished IgM and IgG3 compared to controls (19).

Altogether, these elements are in accordance with the influence of a heterozygous deletion of CARD11, in an immunodeficient phenotype, as in our report.

LFNG (pLI = 0.42) is a key player in the control of NOTCH1 signaling (20). Visan et al. (20) proposed that LFNG and NOTCH1 control progenitor competition for cortical niches that suppress the B-cell potential of progenitors which is important in the regulation of thymus size. Moreover, LFNG is also required for marginal zone (MZ) B cell development (21) and our patients presented a diminution of B memory cells in the marginal zone. LFNG, like PDGFalpha, seems *a priori* well tolerated (pLI = 0.42). This was in agreement with the study of Yu et al. (7). There, they discussed the implication of this gene in the phenotype of their patients, and concluded that haploinsufficiency of only LFNG could not be the cause of the phenotype of these 5 patients.

MafK protein was demonstrated to be critical through its interaction with the transcriptional repressor BTB and CNC homology 2 (Bach2) in repressing the expression of B lymphocyte-induced maturation protein 1 (Blimp-1) (22). However, Blimp-1 expression has to remain low in B cells before increasing in plasma cells. Thus, a misexpression pattern of Blimp-1 could hamper the differentiation of B cells into plasma cells, which could explain the hypogammaglobulinemia M found in our patients. Indeed, the different databases show that MafK is moderately tolerant to LoF (pLI = 0.56). However, such deletion in our patients could have a haploinsufficiency effect on Blimp-1 and generate hypogammaglobulinemia.

An agonist of GPER1 could reduce the enteric macrophage infiltration in a Parkinson's disease mouse model (23).

Very interestingly, genes related to immune deficiency may also have a link with the vasculitis apparition. Indeed, agonists of GPER1 were described to efficiently reduce the expression of TLR4 in macrophages, while knock-down of GPER1 abolished this effect (24). TLR4 expression in dendritic cells was shown to be ubiquitous within all artery types, and involved in pathogen-sensing functions in the innate immune response (25). Thus, a lack of GPER1 may prevent the regulation of TLR4 expression and may facilitate the development of vasculitis.

No CNV has been described for GPER1 in ExAc statistics yet, and DGV polymorphisms mainly concern duplications. GPER1 is expressed in human neutrophils, where it regulates their life span and promotes their activation. This suggests a crucial role for GPER1 signaling in autoimmune and chronic inflammatory diseases in which neutrophils are involved (26). Combined with a high pLI score (0.73), this presents a bundle of arguments indicating an important role of GPER1 in the immune system, and more particularly in the phenotype of patient 1.

Among the 58 genes included in the deletion of our patients, only 5 are described to have an implication (direct or indirect) in the function of immune cells. The analysis of the literature and databases allows us to propose three genes whose role appears to be likely predominant in the patient's 1 phenotype. Functional studies would be necessary to definitively validate their involvement in the onset of the vasculitis episode. Analyses of pLI values highlighted a potential role for GPER-1 (0.73), PDGFalpha (0.86) and especially CARD11 (1), in the immune deficiency, and the cerebral vasculitis observed in Patient 1. Although MafK exhibits a relatively low pLI value (0.56), the implication of this gene in the phenotype of our patients might be explained by the fact that MafK could act indirectly, as a transcriptional repressor in complex with Bach2, to control Blimp-1 expression (whose pLI = 0.98).

Of course, we cannot rule out the involvement of many other polymorphisms (whether or not included in chromosomal rearrangement), since patient 2 expresses a less marked immune phenotype, and has not experienced a vasculitis episode yet. Our patients also exhibit a unique feature with two simultaneous deletions in chromosome 7 and chromosome 15. Although this has not been described yet, we cannot exclude an interaction between these two genome regions, which could also contribute to the clinical presentation of our patients compared to other del7p22 described elsewhere.

## Concluding Remarks

In this clinical report, we highlight a few genes that could, at least in part, explain the complex phenotype of our patients. We hypothesize that (i) the combined deletions of these different genes, all implicated in B cell development and response, and (ii) the combined deletions in chromosome 7 and 15 could explain this phenotype, thus extending the clinical description of these deletions.

## Data Availability

The datasets generated for this study can be found in DECIPHER, 284122.

## Ethics Statement

This study is not exempt of the above requirements, that we all fulfill.

## Author Contributions

NS and BL: clinical examination and editing the manuscript. LL: clinical examination. AS, MV, MB-D, AB, and CBo: biological examinations and interpretation. CBi: neurological clinical examination. FF: hematologic examination, interpretation, and treatment.

### Conflict of Interest Statement

The authors declare that the research was conducted in the absence of any commercial or financial relationships that could be construed as a potential conflict of interest.
